# Back on the Road: Comparing Cognitive Assessments to Driving Simulators in Moderate to Severe Traumatic Brain Injuries

**DOI:** 10.3390/brainsci13010054

**Published:** 2022-12-28

**Authors:** Debra S. Ouellette, Stephanie Kaplan, Emily R. Rosario

**Affiliations:** Casa Colina Hospital and Centers for Healthcare Pomona, Pomona, CA 91769, USA

**Keywords:** driving, cognition, driving simulator, driving assessment, traumatic brain injury

## Abstract

Objective: To compare established clinical outcome assessments for predicting behind the wheel driving readiness and driving simulator results across age groups and in traumatic brain injury. Methods: Participants included adults who had a traumatic brain injury ranging in age from 31 to 57 years and a non-impaired adult population ranging in age from 18 to 80 years. Physical and cognitive outcomes measures were collected included range of motion and coordination, a “Rules of the Road Test” a “Sign Identification Test,” Trails A and B, and the clock drawing test. Visual measures included the Dynavision D2 system and motor-free visual perceptual test-3 (MVPT-3). Finally, the driving simulators (STI^Ò^ version M300) metro drive assessment was used, which consisted of negotiating several obstacles in a metropolitan area including vehicles abruptly changing lanes, pedestrians crossing streets, and negotiating construction zones. Results: Our findings suggest that the standard paper-pencil cognitive assessments and sign identification test significantly differentiate TBI from a non-impaired population (Trails A, B and Clock drawing test *p* < 0.001). While the driving simulator did not show as many robust differences with age, the TBI population did have a significantly greater number of road collisions (F_3, 78_ = 3.5, *p* = 0.02). We also observed a significant correlation between the cognitive assessments and the simulator variables. Conclusions: Paper-pencil cognitive assessments and the sign identification test highlight greater differences than the STI Driving Simulator between non-impaired and TBI populations. However, the driving simulator may be useful in assessing cognitive ability and training for on the road driving.

## 1. Introduction

Driving is an essential activity of daily living for many individuals to maintain their independence [1]. Cessation of driving has often been found to have adverse consequences and has been associated with decreased health related quality of life, depression and social isolation [2]. For patients recovering from injury or illness, return to driving is often identified as a primary goal as it represents independence and autonomy [3,4]. Due to the complexity and heterogeneity of a neurologic injury such as a Traumatic Brain Injury, as well as the lack of clear and consistent clinical guidelines, established outcome measures or parameters it remains very difficult for healthcare providers to determine when an individual is ready to return to driving following the injury [5]. Comprehensive driving evaluations, which include behind the wheel driving, are considered the gold standard for this population [6,7]. However, several clinically validated paper and pencil assessments have been used to help determine readiness to return to on-road driving [8]. Physicians are often reluctant to provide driving clearance to patients due to liability concerns. In a report by the Federal Motor Carrier Safety Administration, they noted an insufficient amount of evidence to address the question of safety for persons with TBI to return to driving [9]. 

Safe return to driving following injury is contingent on cognitive, physical, and visual motor skills [8,9,10,11]. Several assessment tools have been studied in an attempt to develop performance-based neuropsychological predictors of safe driving [12]. The Clock Drawing test has been regarded as a promising screening tool of driving performance, as it is a brief and easy to administer test [13,14]. Another cognitive measure that has been found to correlate highly with driving performance is the Trail Making Test A and B, which assesses the domain of visual conceptual and visuomotor [15,16]. Further, both the clock drawing test and Trails A and B have been shown to be good predictors of driving performance using a driving simulator [13,17,18]. The Motor Free Visual Perceptual Test 3 (MVPT 3), which assesses the domain of visual perceptual ability, has also been shown to predict an increased likelihood of on-road accidents and it correlates with driving performance in stroke populations [19,20]. Persons who had poor performance on the cognitive domain of Trail Making B and MVPT where 22 times more likely to fail on-road evaluation [21,22]. Finally, the Dynavision, which assesses the domains of visual scanning, peripheral visual awareness, visuomotor reaction time, and basic cognitive skills and physical/mental endurance, has been used as a successful clinical tool in predicting success/failure in the on-road driving test [23,24]. 

The use of driving simulators is becoming more common by both occupational therapists and researchers to predict readiness for on the road driving and to understand underlying factors involved with safe driving and crash avoidance [25,26]. Driving simulators are able to provide stimulus control, allowing for controlled data collection verses on the road assessment in which factors are more dynamic and variable each time. Multiple studies have found driving simulators useful in the evaluation of on-road driving abilities or fitness to drive in older individuals, those with Parkinson’s, stroke, and brain injury [5,25,27,28]. Specifically, evidence suggests driving simulators are able to detect differences between young and aging drivers on variables such as speed, steering control, divided attention, lane boundary crossing, and total collisions [25,29]. Driving simulators have also been reported to detect differences in driving ability on the above variables and others be-tween healthy drivers and those with neurologic injury including traumatic brain injury (TBI [5,27] stroke [22,30], Parkinson’s Disease (PD) [27,31], Multiple Sclerosis [32], and dementia [13,33]. 

Despite the current utility of neuropsychological cognitive, clinical pre-driving assessments and simulator assessments, there is a lack of comparison of these outcomes and changes that over with age or difference between impaired and non-impaired populations. Therefore, the purpose of this study to compare clinically proven outcome assessments for predicting behind the wheel driving readiness and driving simulator results in across age groups and in traumatic brain injury. 

## 2. Materials and Methods

### 2.1. Participants

A sample of 62 healthy adults ranging in age from 18–80 years was recruited from a random sample of staff and individuals from the community. A second sample of 21 traumatic brain injury patients from age 31 to 57 years were recruited from a rehabilitation facility. Exclusion for the non-impaired population included: not having a valid driver’s license, a history of a previous brain injury, inability to read and not having proficiency in the English language. Participants for the non-impaired population were assigned to a group based on age (18–40, 41–65, and 66–85). The exclusion for the TBI population was inability to understand the directions, not having proficiency in the English language, inability to read and follow directions.

### 2.2. Study Design

Prior to initiation of this study, it was reviewed and approved by the Institutional Review Board and all research was done in accordance to required ethical standards. Study assessments were conducted in a single two-hour session by an occupational therapist, physical therapist or research assistant. Each was trained in administering the assessments prior to independent administration for each participant. Participants first completed self-reported intake forms that documented prior medical history, driving history, pain and dizziness, (both pain and dizziness were on a zero-to-ten-point scale, with ten indicating the most severe symptoms, the dizziness and pain scales would also be noted post simulator use).

### 2.3. Outcome Measures

Physical measures included active assessment of bilateral upper extremity, lower extremity, and trunk range of motion, muscle strength, and coordination to establish if a participant would physically be able to use the driving simulator. 

Cognitive/Visual measures included the “Rules of the Road Test” (Max 5), the “Sign Identification Test” (max 4) (both based on the written driving test given by the state of California Department of Motor Vehicles), the Trails Making A and B test, the Clock Drawing test, Dynavision D2, and the Motor-Free Visual Perceptual Test-3 (MVPT-3).

The Systems Technology Incorporated Simulator (STI version M300) (STISIM) driving simulator is a PC based interactive simulator that is designed to immerse the participant in a range of psychomotor and cognitive tasks. The driving tasks that were selected for use in this study included: introduction to traffic violation (D) assessment and metro drive (D) assessment, which included 39 variables. 

### 2.4. Statistical Analysis

The mean and standard deviation were reported according to age groups. Differences between the TBI population and the 3 non-impaired age groups were assessed with an ANOVA and correlations were run to compare the paper pencil and Dynavision assessments with the driving simulator. 

## 3. Results

### 3.1. Participants

The non-impaired population was binned into one of 3 age-range categories, 18–40 (*n* = 21), 41–65 (*n* = 23), and over 65 (*n* = 18). The mean age overall for the non-impaired population was 50.3 ± 19.2 years while the mean age for each non-impaired population age group was 28.95 ± 6.6, 50.9 ± 7.3, and 74.3 ± 5.5 years, respectively. The mean age for the TBI population was 44.14 ± 13.2 years (Table 1). When comparing medical information across all groups, medical issues such as diabetes and hypertension increased with age, however vertigo, seizures, loss of consciousness, and dizziness had the highest incidence in the TBI population (Table 1). 

### 3.2. Driving Information

Driving information provided by the participants was consistent with national driving statistics in that the 65–85-year-olds spend less time driving on freeways (88%), at night (83%), and long distances (72%) (Table 2). The TBI population followed by the 18–40-year-olds had the greatest number of moving violations and accidents. A significant difference in the number of accidents was noted between the groups with the 41–64 years having the fewest (0.04) compared with the 18–40 (0.38), 65+ (0.28), and the TBI population (0.71) (Table 2). All ages scored similarly on the rules of the road and sign identification tests, except the TBI population had significantly greater impairment on the sign identification tests (F (3, 78) = 15.7, *p* < 0.001).

### 3.3. Congitive Function and Visual Perception

Results for the cognitive function and visual perception tasks showed expected age-related changes and differences in the TBI population (Table 3). A significant increase in the time taken to complete Trails A and B was observed in the TBI population and with the 65+ group (F (3, 74) = 9.9, *p* < 0.001; F (3, 74) = 14.4, *p* < 0.001). The clock-drawing test showed significant differences in cognition for the TBI population (F (3, 74) = 11.0, *p* < 0.001). The Dynavision D2 Mode B with flash showed significant age-related differences while the TBI population performed similar to the 18–40-year-olds (F (3, 74) = 9.9, *p* < 0.0001). Similarly, when looking at visual perception with the MVPT-3 we observed significant differences between the 41–65-year-olds and 65–85-year-olds and 18–40-year-olds and the TBI population (Table 3).

### 3.4. Driving Simulator

Results with the driving simulator only showed one significant age-related difference. Interestingly, the TBI population had a significantly greater number of road collisions (Table 4, F (3, 78) = 3.5, *p* = 0.02). When looking at age-related changes the 18–40-year-olds trended towards having the greatest number of collisions, traffic tickets, and the slowest reaction time overall. Spearman rank correlations were done to determine if the driving simulator data correlates with any of the medical, driving, or clinical assessment data that were collected. Significant correlations were observed between the simulator, reaction time and cognitive abilities (Table 5). Specifically, we found cognitive impairments were associated with the percent of time outside of the lane and time over the speed limit. The majority of these correlations were also seen in the 65+ group but not the other age groups. The exception was with the Dynavision mode B, where a significant correlation was observed between percentage of time spent outside of the lane and the number of hits. This correlation was also present in the 18–40-year-old group (Table 5). Using the MVPT-3 as a visual perception assessment we found significant correlations between the MVPT-3, number of collisions, percent of time outside of the lane and number of times over the speed limit (Table 5). When correlations with the MVPT were run for each individual age group these correlations were no longer significant. Finally, we saw interesting relationships between the five-point rules of the road test and driving metrics on the simulator including number of traffic tickets, time outside of the lane and time over the speed limit. When these correlations were run for the different age groups, we found relationships for the 65+ and 18–40-year-old groups only.

## 4. Discussion

Driving is an essential activity for independent living and often identified as a goal for patients who are recovering from injury or illness. Safe return to driving following injury is contingent on both cognitive and physical status however, clear and consistent clinical guidelines to determine when an individual is ready to return to driving following an injury are lacking. While a review of the literature on TBI and Driving assessment has concluded there is insufficient evidence that a driving simulator can substitute for real-world driving [17,34], there appears to be the potential for stimulator use for rehabilitation intervention and assessment [5]. The purpose of this study was to compare clinically proven outcome assessments for predicting behind the wheel driving readiness and driving simulator results in across age groups and in traumatic brain injury. 

Our study found that paper-pencil cognitive assessments and sign identification strongly identify significant differences between individuals with TBI and the non-impaired population; however, the addition of driving simulator data did not further stratify these groups.

The results of this study replicate the work of other researchers who have found a correlation between neuropsychological tests and driving skill. The older group and the TBI population in this study was noted to have lower scores on the cognitive measures (Trails A, B and the clock test), which have been shown to be a salient predictor of crash involvement in older adult [19]. The Clock Drawing test which assesses executive function, has been found to have limited utility as a solitary measure of on-road performance for patients with dementia, but has been suggested to be useful when paired with other screening assessments [35]. In our study the Clock test showed significant differences between the TBI group and all of the non-impaired groups, with decreased executive function in the TBI group population. Previous studies have also found that persons who had poor performance on the cognitive domain of Trail Making B and MVPT where 22 times more likely to fail an on-road evaluation [22]. In our study both Trails A and B showed significant differences when comparing the TBI and the non-impaired groups. Interestingly, the MVPT-3 showed a significant difference between groups however the TBI group performed similar to the 18–40-year-olds suggesting more age-related changes than injury related impairment. We found a similar pattern with the Dynavision Mode B with similarities in performance between the 18–40-year-olds and the TBI population and a significant difference in performance between these groups and the 41–65- and 65–85-year-old non-impaired groups (*p* < 0.0001, F (3, 74) = 9.9). Our data is in contrast to previous studies, which has shown the Dynavision to be a successful clinical tool in predicting success/failure in the on-road driving test [23,24]. One difference between our study and the previous studies was the number of trials or practice that was given [24]. In a recent study it was found that 15 training sessions were needed to overcome produce a reliable performance for the test [36].

Results with the driving simulator are in line with earlier reports regarding younger drivers as we found that the 18–40-year-old group showed an increase in collisions, number of traffic light tickets, and number of times over the speed limit, which may be related to increased risk taking. Younger drivers between the ages of 18 to 25 years old are more likely to run red lights as compared to other age groups [37]. This finding is also supported by earlier studies that noted red light runners tend to be drivers under 30 [38,39]. Interestingly, we also found that the youngest group showed increased pedal reaction time (taking a longer time to engage the brake pedal when slowing down), which may suggest a lack of attention to the task, distraction while driving, or a lack of experience which results in misestimation of the time it takes to cover a set distance. We only observed 1 significant effect for the TBI population in all the variables measured with the driving simulator. While the TBI population had the greatest number of road collisions with the simulator, a significant difference was only observed when comparing with the 41–65 and 65–85-year-olds. A significant difference between the TBI and 18–40-year-old non-impaired groups was not observed. Studies have found that the strategy and movements made in a simulator are similar to actual driving [40]. However, the level of visual engagement was lower in a simulator [40]. Another study found that a when a physiological response was measured a stress reaction and higher level of arousal was only measured in the real vehicle [41].

There are advantages with the driving simulator which include a safe environment to observe individuals driving behaviors in a virtual reality version of real road situations, direct and real time feedback to the participant of their performance via the driving simulator, and driving simulators are found to have higher face validity than pencil paper tests for pre-on road assessment [5]. In addition, patients anecdotally accept the feedback from their performance on the driving simulator more readily due to the real time feedback and look forward to additional opportunities to practice. However, in this study paper-pencil cognitive assessments and the sign identification test appear to be the best predictors of both TBI related impairments and behind the wheel driving readiness. Use of the STI Driving Simulator alone or in conjunction with other tests and measures was not as strong of predictor as anticipated but as mentioned above may have a role in aiding the return to driving following TBI. More research is also needed to see if practice sessions would help to improve the validity of the testing measures.

### Study Limitations

The study was limited by a convenience sample along with a smaller sample size. We had participants who began the simulator assessment but showed Simulator Adaptation Syn-drome (SAS) and had to modify the protocol. SAS includes nausea, sweating, headaches, disorientation, vertigo, and eyestrain [42]. This can affect the client’s attention and driving performance as well as the validity of the simulator outcome [43]. To counteract SAS the two side screens were turned off for those individuals, which modified the visual input of trees, mountains on the periphery. All of the other content remained the same. 4.8% of participants required the side screens turned off.

## 5. Conclusions

The focus of this study was to assess pencil paper assessments with driving simulator data to determine if the use of the driving simulator in conjunction with the standardized paper pencil test assists clinicians in predicting behind the wheel driving readiness for a traumatic brain injury population. Our findings suggest that paper-pencil cognitive assessments and sign identification tests do the best at differentiating TBI from a non-impaired population. When using the driving simulator TBI patients perform similar to the 18–40-year-old group of non-impaired individuals. It has been noted in best practice guidelines for driving that the driving simulator cannot be the sole determinant of fitness to drive in older adults, but the simulator activities may be valuable as part of a comprehensive driving evaluation [5,6].

As driving simulators are becoming more common in the field of rehabilitation our data suggests that the addition of a driving simulator does not further define differences between a non-impaired and TBI population, but may be used as an adjunct to on the road driving.

## Figures and Tables

**Table 1:** Demographic/Medical Intake information (self-reported).

Demographic/Medical Information	18–40 Year Olds (*n* = 21)	41–65 Year Olds (*n* = 23)	65–85 Year Olds (*n* = 18)	TBI Population (*n* = 21)
Age (Mean **±** SD)	28.95 ± 6.6	50.9 ± 7.3	74.3 ± 5.5	44.14 ± 13.2
Gender	81% F/19% M	65% F/35% M	50% F/50% M	15% F/85% M
Ave # of med issues	0.48 ± 0.2	0.70 ± 0.2	1.3 ± 0.2 *	N/A
Medical Issues	85% No	48% No	22% No	N/A
Seizures	95% No	100% No	88% No	86% No
Loss of Consciousness	90% No	91% No	94% No	24% No
Dizziness	90% No	95% No	83% No	43% No
Vertigo	95% No	91% No	83% No	66% No
Diabetes	100% No	95% No	78% No	95% No
Hypertension	81% No	56% No	44% No	71% No
Pain level (1–10) (mean score)	0.14 + 0.48	0.14 + 0.64	0.4 + 0.74	0.86 + 1.65 *

* denotes significance at *p* < 0.05. # denotes number.

**Table 2:** Intake Driving information.

Driving Information	18–40 Year Olds (*n* = 21)	41–65 Year Olds (*n* = 22)	65–85 Year Olds (*n* = 15)	TBI Population (*n* = 21)
Automatic transmission	100%	91%	93%	86%
Drive on freeway	90%	100%	88%	95%
Drive at night	100%	100%	83%	95%
Drive long distance	86%	100%	72%	95%
Drive for work	100%	91%	33%	71%
# of moving violations (Mean)	0.48 ± 0.75	0.22 ± 0.4	0.05 ± 0.5 *	0.57 ± 0.87
# of Accidents (Mean)	0.38 ± 0.5	0.04 ± 0.2 *	0.28 ± 0.11	0.71 ± 1.4
Rules of the road (max 5)	4.5 ± 1	4.95 ± 0.2	4.6 ± 0.8	4.35 ± 0.93
Sign Identification (4 signs)	3.9 ± 0.5	4 ± 0	3.94 ± 0.2	3.15 ± 0.75 *

* denotes significance at *p* < 0.05. # denotes number

**Table 3:** Clinical Assessments.

Assessments	18–40 Year Olds (*n* = 21)	41–65 Year Olds (*n* = 24)	65–85 Year Olds (*n* = 17)	TBI (*n* = 16)	ANOVA
Trails A	24.4 ± 2.4 ^C^	27.9 ± 2.3 ^BC^	38.3 ± 2.6 ^B^	46.3 ± 3.2 ^A^	*p* < 0.0001 F (3, 74) = 9.9
Trails B	57.6 ± 6.6 ^B^	58.3 ± 6.3 ^B^	94.6 ± 7.1 ^B^	170.0 ± 14.3 ^A^	*p* < 0.0001 F (3, 74) = 14.4
Clock test 6–1	5.4 ± 0.14 ^B^	5.7 ± 0.14 ^B^	5.2 ± 0.15 ^B^	2.8 ± 1.6 ^A^	*p* < 0.0001 F (3, 74) = 11.0
Dynavision, Mode A ave. rxn time	0.99 ± 0.04	1.13 ± 0.04	1.4 ± 0.04	1.3 ± 0.12	*p* = 0.23 F (3, 74) = 1.46
Dynavision, Mode B with Flash # hits	37.0 ± 2.7 ^A^	26.0 ± 2.6 ^B^	13.3 ± 3.0 ^C^	30.9 ± 3.1 ^A^	*p* < 0.0001 F (3, 74) = 9.9
Motor free Visual Perception (SS)	99.3 ± 4.2 ^B^	123.4 ± 3.9 ^A^	117.0 ± 4.3 ^A^	96.8 ± 4.3 ^B^	*p* = 0.0001 F (3, 79) = 9.5

Data represents the mean ± SEM. # denotes number. Groups not connected by the same letter are significantly different, i.e., A is statistically different from B and C and B is statistically different from A and C.

**Table 4:** Driving simulator data.

Driving Simulator Data (Hazard Perception)	18–40 Year Olds (*n* = 21)	41–65 Year Olds (*n* = 22)	65–85 Year Olds (*n* = 15)	TBI (*n* = 16)	*p* Value
# of road collisions	0.64 ± 0.12	0.35 ± 0.11	0.31 ± 0.14	0.8 ± 0.14 *	*p* = 0.02 F (3, 78) = 3.5
# of traffic light tickets	0.19 ± 0.06	0.0 ± 0.06	0.11 ± 0.07	0.04 ± 0.07	*p* = 0.14 F (3, 78) = 1.8
# of times over speed limit	2.4 ± 0.36	1.68 ± 0.4	2.4 ± 0.4	2.4 ± 0.4	*p* = 0.53 F (3, 78) = 0.7
# of times driver went off road	0.7 ± 0.09	0.7 ± 0.08	0.94 ± 0.1	0.9 ± 0.09	*p* = 0.17 F (3, 78) = 1.7
% of time outside of lanes	0.2 ± 0.2	0.25 ± 0.2	0.92 ± 0.2	0.26 ± 0.2	*p* = 0.14 F (3, 78) = 1.8
Total pedal reaction time	13.26 ± 2.4	6.6 ± 2.4	4.9 ± 2.7	12.6 ± 2.7	*p* = 0.06 F (23, 78) = 2.5

* denotes significance at *p* < 0.05. # denotes number. % denotes the percentage.

**Table 5:** Correlations between simulator data and Clinical assessment data.

Driving Simulator Data	Clinical Assessment Data	Spearman Rank ρ
	***Attention/Cognition***	
% of time out of lane	Trails A ^1^	0.377 **
Total # of times over speed limit	Trails A ^1^	0.2583 *
% of time out of lane	Mode A average reaction time	0.2732 *
% of time out of lane	Mode B with flash #hits ^2^	−0.3147 *
	***Visual Perception***	
% of time out of lane	MVPT standard score	−0.2657
Total # of times over speed limit	MVPT standard score	−0.3794
Total # of collisions	MVPT standard score	−0.3649
	***Driving assessment***	
% of time out of lane	Rules of road/5 ^2^	−0.4433
Total # of times over speed limit	Rules of road/5 ^1,2^	−0.3624
Total # of traffic light tickets	Rules of road/5 ^1^	−0.2819

* *p* < 0.05, ** *p* < 0.01; ^1^ when analyzed by age bin a significant correlation was present for the 65+ group; ^2^ when analyzed by age bin a significant correlation was present for the 18–40-year-old group. # denotes number.

## Data Availability

All data that support the findings of this study are available upon request from the corresponding author.
